# The role of REBOA in patients in traumatic cardiac arrest subsequent to hemorrhagic shock: a scoping review

**DOI:** 10.1007/s00068-022-02154-z

**Published:** 2022-11-06

**Authors:** S. A. S. Slot, S. E. van Oostendorp, L. J. Schoonmade, L. M. G. Geeraedts

**Affiliations:** 1 Amsterdam UMC, Location VUMC, Department of Surgery, Section Trauma Surgery, Vrije Universiteit Amsterdam, De Boelelaan 1117, P.O. Box 7057, 1007 MB Amsterdam, The Netherlands; 2 Department of Surgery, Rode Kruis Ziekenhuis, Beverwijk, The Netherlands; 3 Medical Library, Vrije Universiteit Amsterdam, De Boelelaan 1117, P.O. Box 7057, 1007 MB Amsterdam, The Netherlands

**Keywords:** REBOA, Traumatic cardiac arrest, Resuscitative endovascular balloon occlusion of the aorta, Hemorrhagic shock

## Abstract

**Purpose:**

Resuscitative endovascular balloon occlusion of the aorta (REBOA) is a useful adjunct in treatment of patients in severe hemorrhagic shock. Hypothetically, REBOA could benefit patients in traumatic cardiac arrest (TCA) as balloon occlusion of the aorta increases afterload and may improve myocardial performance leading to return of spontaneous circulation (ROSC). This scoping review was conducted to examine the effect of REBOA on patients in TCA.

**Methods:**

This scoping review was conducted using the Preferred Reporting Items for Systematic Reviews and Meta-Analysis Extension for Scoping Reviews (PRISMA-ScR) Statement. PubMed, EMBASE.com and the Web of Science Core Collection were searched. Articles were included if they reported any data on patients that underwent REBOA and were in TCA. Of the included articles, data regarding SBP, ROSC and survival were extracted and summarized.

**Results:**

Of 854 identified studies, 26 articles met criteria for inclusion. These identified a total of 785 patients in TCA that received REBOA (presumably less because of potential overlap in patients). This review shows REBOA elevates mean SBP in patients in TCA. The achievement of ROSC after REBOA deployment ranged from 18.2% to 67.7%. Survival to discharge ranged from 3.5% to 12.1%.

**Conclusion:**

Overall, weak evidence is available on the use of REBOA in patients in TCA. This review, limited by selection bias, indicates that REBOA elevates SBP and may benefit ROSC and potentially survival to discharge in patients in TCA. Extensive further research is necessary to further clarify the role of REBOA during TCA.

**Supplementary Information:**

The online version contains supplementary material available at 10.1007/s00068-022-02154-z.

## Introduction

Old trauma resuscitation interventions have their way of being resuscitated themselves: tourniquets, for example, have been rediscovered and currently play a major role in preventing exsanguination from extremity injuries. However, hemorrhage locations such as intra-abdominal or intra-thoracic remain more difficult to manage in the pre-hospital situation due to their non-compressible nature. Truncal hemorrhage accounts for the majority of potentially preventable fatalities in trauma patients [1, 2]. Ninety percent of deaths caused by hemorrhage are truncal, while extremity hemorrhage account for the remaining 10% of traumatic bleeding mortality [3]. Excessive, non-compressible truncal bleeding can quickly lead to decreased circulatory volume, cardiac arrest and eventually death. Emergency thoracotomy (ET) can be performed as an ultimate attempt for a life-saving procedure for patients with such an injury. This enables cross-clamping of the aorta and direct cardiac massage. However, ET is a highly invasive procedure with a mortality rate of above 90%, reflecting the extremis in which these patients arrive [4]. As with the tourniquet, a relatively old procedure has come to light as an alternative to ET: resuscitative endovascular balloon occlusion of the aorta (REBOA). REBOA was first described by Hughes et al. during the Korean War in 1954 and consists of a balloon, catheter and guidewire that can be accessed via the CFA (common femoral artery) to be inflated in the aorta [5]. By stopping aortic perfusion distally, bleeding can be temporarily controlled. Nowadays, REBOA is used in patients in hemorrhagic shock, also for non-traumatic indications such as aortic aneurysm and postpartum hemorrhage, and may function as a temporary bridge to definitive control by surgery or interventional radiology [6–10]. It has a reported lower procedure-related morbidity compared to ET, yet complications such as lower leg amputation, intestinal ischemia and kidney failure due to prolonged occlusion have been reported [11]. Limited evidence is available on the use of REBOA in patients in traumatic cardiac arrest. Physiologically, occluding the aorta increases blood pressure in the aorta proximal to the occlusion as well as cardiac afterload and therefore augments coronary perfusion. In addition, several studies on humans and animals in traumatic and non-traumatic cardiac arrest have proven that higher coronary pressures augment the chance of return of spontaneous circulation (ROSC) [12–14]. A recent statement from the American College of Surgeons Committee on Trauma confirms REBOA is indicated in patients in traumatic cardiac arrest, specifically during the time period one would normally perform ET [15]. The aim of this scoping review is therefore to examine whether REBOA provides a survival benefit to massive exsanguinating patients in traumatic cardiac arrest and to shed light on the effect of REBOA on important hemodynamic parameters such as systolic blood pressure (SBP) and the occurrence of ROSC.

## Method

### Search methods

This scoping review was reported in accordance with the Preferred Reporting Items for Systematic Reviews and Meta-Analysis Extension for Scoping Reviews (PRISMA-ScR) Statement [16]. A comprehensive search was performed in the bibliographic databases PubMed, Embase.com and Web of Science Core collection from inception to September 15, 2021, in collaboration with a medical librarian (LS). Search terms included controlled terms (MeSH in PubMed and Emtree in Embase) as well as free text terms. The following terms were used (including synonyms and closely related words) as index terms or free-text words: ‘REBOA’ and ‘resuscitation’ and ‘endovascular’ and ‘aorta.’ The search was performed without date or language restrictions. The full search strategies for all databases can be found in the Supplementary Information.

### In- and exclusion criteria

Articles were included if they incorporated (a group of) patients in traumatic cardiac arrest that underwent REBOA and if they reported on mortality and hemodynamics in this group of patients. If the article stated patients had asystole, PEA (pulseless electrical activity) or were receiving CPR after trauma, this was considered TCA. Studies where no REBOA was used, animal studies, studies regarding non-traumatic injuries, systematic reviews, conference abstracts and letters were excluded. Papers in any other language than English were excluded as well.

### Selection process

**Fig. 1:**
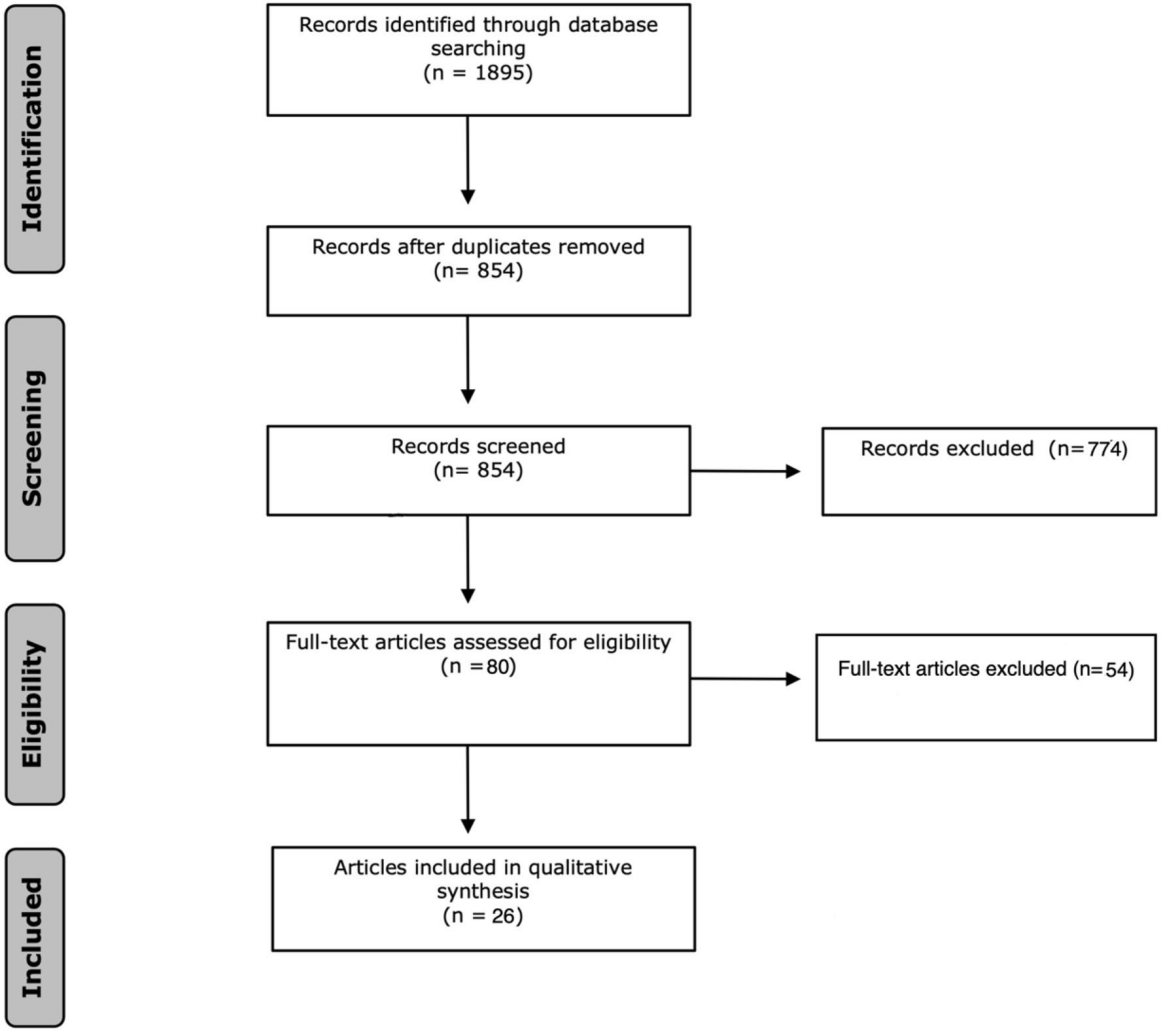
Flowchart of the search and selection procedure of studies

Duplicate articles were excluded by a medical information specialist (LS) using Endnote X20.0.1 (Clarivate^tm^), following the Amsterdam Efficient Deduplication (AED)-method and the Bramer-method [17, 18]. Two reviewers (SS and SvO) independently screened all potentially relevant titles and abstracts for eligibility using Rayyan [19]. The remaining articles were assessed for eligibility by full-text reading by the previously mentioned independent reviewers. The flowchart of the search and selection process is presented in Fig. 1. Differences in judgment were resolved through a consensus procedure. All levels of evidence were considered. Of the final chosen papers, data regarding SBP, ROSC and survival were extracted and summarized. No quantitative analysis was possible due to heterogeneity of study design and data. The case reports were not included in the reported ranges of the results section, yet can be found in the summarizing table.

## Results

The literature search generated a total of 1895 references: 566 in PubMed, 681 in Embase and 648 in Web of Science. After removing duplicates of references that were selected from more than one database, 854 references remained. Eighty articles were included for a full-text analysis. After resolving possible conflicts, 26 papers met the inclusion criteria and were found eligible for the study. Year of publication from the included studies ranged from 2013 to 2021. Thirteen studies were performed in the USA, of which nine in the Shock and Trauma Center in Baltimore, Maryland, USA on adult patients and could therefore enfold a potential overlap in patients. Nevertheless, these articles were included because they all commented on different outcome measures that were interesting to describe in this review. One study with data from the Shock and Trauma Center in Baltimore described the outcome of REBOA in a cohort of pediatric patients in TCA and therefore forms no overlap. Three studies were performed in Japan, one in Korea, one in Italy and one in Sweden. The data of the remaining seven studies was extracted from three different international registries.

**Fig. 2:**
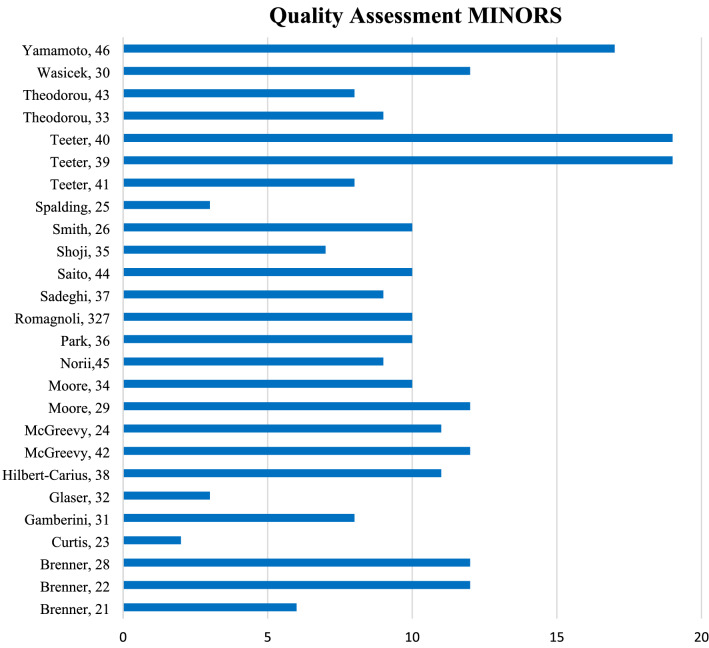
Quality assessment of all included articles using the Methodological Index for Non-Randomized Studies (MINORS)

The 26 included studies provided data of 785 patients, note non-unique due to suspect of overlap, in traumatic cardiac arrest that received REBOA were included in the study. Main results regarding SBP improvement, ROSC and survival of TCA patients are shown in Table 1. This table also shows the institution or database the study was performed at Fig. 2 shows a quality assessment of all included papers using the Methodological Index for Non-Randomized Studies (MINORS) [20]. Ideally, this score is 16 for non-comparative and 24 for comparative studies. In this review, the average MINORS-score for all studies was 9.9. Excluding case reports, the MINORS-score was 11.2.

**Table 1:** Summary of all included articles

Refs.	Author	Year	Institution or database	Inclusion criteria	Study type	Patients (total)	Patients in TCA + REBOA	Median ISS (total)	Median ISS (TCA + REBOA)	SBP (during TCA + REBOA)	ROSC	Survival (in TCA patients, short term)	Survival (in TCA patients, 24 h)	Survival (in TCA patients, to discharge)	Specific statement REBOA was inflated during arrest
[21]	Brenner^a^	2013	Adams Cowley Shock Trauma Center Baltimore, Maryland and Herman Memorial Hospital Houston, Texas, USA (December 2012 to March 2013)	Patients in end-stage hemorrhagic shock receiving REBOA	Case series	6	*1*	NR	9	SBP improved from 0 to 100	*100%*	*100% (no time stamp)*	NR	NR	Yes
[22]	Brenner^a^	2018	Adams Cowley Shock Trauma Center Baltimore, Maryland, USA (February 1 2013 to January 31 2017)	Patients that received REBOA for traumatic hemorrhage, arrest, and non-traumatic hemorrhage	Prospective observational	90	50	39	NR	Mean SBP improved from 15.1 to 71.0	58%	40% (8 h)	NR	10% (30 day)	Yes
[28]	Brenner^a^	2019	Adams Cowley Shock Trauma Center Baltimore, Maryland and Herman Memorial Hospital Houston, Texas, USA (February 2016 to February 2017)	Patients receiving AO with the ER-REBOA device	Prospective observational	60	31	NR	NR	NR	67,70%	19% (in-hospital)	NR	NR	Yes
[23]	Curtis	2019	University of California Davis Medical Center, Sacramento, USA	None	*Case report*	*1*	*1*	NR	NR	SBP improved from 45 to above 100	*100%*	*100% (no time stamp)*	NR	NR	Yes
[31]	Gamberini	2021	Maggiore Hospital Carlo Alberto Pizzardi, Italy (January 2019 to December 2020). Systems Saving Lives database	Patients in OHCA in whom REBOA was attempted	Case series	20	7	NR	NR	NR	42.9%	0% (in-hospital)	NR	NR	Yes
[32]	Glaser	2018	Naval Medical Research Unit San Antonio, Texas, USA. Grand Strand Medical Center, Myrtle Beach, South Carolina, USA	None	Case series	7	3	NR	NR	NR	NR	0% (no time stamp)	NR	NR	Yes
[38]	Hilbert-Carius^b^	2020	ABOTrauma Registry (2014 to 2019)	TCA, pre-hospital CPR, RISC-II score, availability of outcome data, REBOA performed early after admission	Retrospective and prospective observational	26	8	45,5	NR	NR	NR	14% (no time stamp)	NR	NR	Yes
[42]	McGreevy	2021	Örebro University Hospital, Sweden (October 2015, May 2020)	Patients in any type of hemorrhagic shock that received the ER-REBOA catheter	Prospective observational	22	12	NR	NR	NR	75%	NR	40%	NR	No
[24]	McGreevy^b^	2020	ABOTrauma Registry (retrospectively from November 2011, prospectively September 2014 to January 2019)	Patients that received REBOA in traumatic hemorrhagic shock	Retrospective and prospective observational	74	74	41	41	Median post-inflation SBP improved to 90	NR	36.6% (no time stamp)	NR	NR	Yes
[29]	Moore^a^	2020	Six different level 1 Trauma Centers, USA (May 2017 to June 2018)	> 15 years, truncal hemorrhage, control within 60 min of arrival	Prospective observational	75	17	34	NR	NR	58.50%	NR	NR	5.80%	Yes
[34]	Moore	2016	Texas Trauma Institute, Houston, USA (October 2011 to September 2015)	Any patient that received a REBOA procedure in the acute phase after injury	Retrospective case series	31	10	34	NR	NR	60%	NR	NR	10%	Yes
[45]	Norii^c^	2019	Japan Trauma Data Bank (2004 to 2015)	All adult patients that experienced blunt TCA	Retrospective observational	8347	175	34	NR	NR	NR	NR	NR	5.10%	Yes
[36]	Park	2019	Gachon University Gil Medical Center, Incheon City, South-Korea (December 2015 to January 2019)	Trauma patients that underwent REBOA	Retrospective observational	24	10	36,2	NR	NR	NR	0% (no time stamp)	NR	0%	No
[27]	Romagnoli^a^	2018	University of Maryland School of Medicine, Baltimore, Maryland, USA (May 2014 to September 2017)	All adult patients that underwent REBOA and were videotaped	Retrospective observational	74	51	37,45	NR	NR	62.70%	NR	NR	9,80%	Yes
[37]	Sadeghi^b^	2017	ABOTrauma registry (November 2011to September 2016)	Patients in traumatic shock that underwent REBOA	Retrospective and prospective observational	96	11	41	NR	NR	NR	27% (no time stamp)	NR	NR	No
[44]	Saito	2014	Shock and Trauma Center of Nippon Medical School Chiba Hokusoh Hospital, Japan (January 2007 to December 2013)	Adult patients that underwent REBOA	Retrospective observational	24	3	47	NR	NR	NR	0% (in-hospital)	NR	NR	No
[35]	Shoji	2017	Kyorin University Hospital Tokyo, Japan (June 2014 to September 2016)	Patients in all-cause hemorrhagic shock that underwent REBOA	Retrospective observational	10	*1*	NR	NR	NR	*100%*	*0% (no time stamp)*	NR	NR	Yes
[26]	Smith	2020	Adams Cowley Shock Trauma Center Baltimore, Maryland, USA (August 2013 to February 2017)	Pediatric patients that underwent REBOA	Retrospective observational	7	4	36	NR	SBP post inflation ranged from 0 to 160	100%	NR	NR	0%	No
[25]	Spalding	2018	Grant Medical Center, Ohio, USA	None	*Case report*	*1*	*1*	NR	NR	SBP improved from 0 to 160	*100%*	*100% (no time stamp)*	*100%*	*100%*	Yes
[41]	Teeter	2016	Five tertiary-care hospitals in Japan (January 2014 to June 2015)	Patients that arrived to the ER in cardiac arrest and underwent REBOA or RT after	Retrospective observational	33	10	38	NR	NR	NR	NR	20%	NR	No
[39]	Teeter^a^	2018	Adams Cowley Shock Trauma Center Baltimore, Maryland, USA (February 2013 to September 2016)	Patients that arrived to the center in cardiac arrest and underwent OCCM during REBOA or ACC	Retrospective and prospective observational	51	33	NR	37	NR	60.10%	45.4% (past ER/OR)	NR	12,10%	Yes
[40]	Teeter^a^	2018	Adams Cowley Shock Trauma Center Baltimore, Maryland, USA (May 2014 to December 2016)	All adult trauma patients that underwent REBOA	Prospective observational	50	22	27	34	NR	NR	40.5 (past ER)	NR	9%	Yes
[33]	Theodorou	2020	AAST AORTA registry (November 2013 to January 2018)	All adult trauma patients that underwent REBOA	Retrospective observational	271	92	34	NR	NR	NR	8.7% (in- hospital)	NR	NR	Yes
[43]	Theodorou	2021	AAST AORTA registry (September 2013 to April 2020)	All pediatric trauma patients that underwent REBOA	Retrospective observational	11	3	29	NR	NR	66.60%	NR	NR	NR	Yes
[30]	Wasicek^a^	2018	Adams Cowley Shock Trauma Center Baltimore, Maryland, USA (February 2013 to May 2017)	Patients in TCA, had an arterial line placed during resuscitation and received REBOA and CPR	Retrospective and prospective observational	58	11	NR	NR	NR	18.20%	36% (12 h)	27%	NR	Yes
[46]	Yamamoto^c^	2020	Japan Trauma Data Bank (January 2004, March 2019)	Patients with t-OHCA, aged > 15 years, who had arrived without a palpable pulse and with a GCS > 3, who received AO through RT or REBOA	Retrospective observational	1483	144	NR	36	NR	NR	NR	NR	3.50%	Yes
							785							26	

Results from case reports or series with only one patient are shown in italic

*NR* not reported; *TCA* traumatic cardiac arrest; *ROSC* return of spontaneous circulation; *GCS* Glasgow Coma Scale; *ER* emergency room; *OR* operation room; *SBP* systolic blood pressure; *CPR* cardiopulmonary resuscitation; *t-OHCA* traumatic out of hospital cardiac arrest; *REBOA* resuscitative endovascular balloon occlusion of the aorta; *RT* resuscitative thoracotomy; *ACC* aortic crossclamping; *OCCM* Open Chest Cardiac Massage

^a^Potential overlap in patients from Adams Cowley Shock Trauma Center Baltimore, Maryland, USA

^b^Potential overlap in patients from ABOtrauma registry

^c^Potential overlap in patients from Japan Trauma Data Bank

### Efficacy

#### SBP

Of the 26 included studies describing patients in traumatic cardiac arrest, six studies commented on systolic blood pressure during REBOA deployment [21–26]. All six articles stated a significant improvement in systolic blood pressure after balloon inflation. Important to highlight is that two studies by Brenner et al. were performed in the same center and could have a potential overlap of patients [21, 22]. Two case reports specified pressure was measured by continuous registration through arterial line placement or aortic pressure through the REBOA catheter itself [21, 23, 25]. Others were non-specific or did not report on the manner of blood pressure measurement.

#### ROSC

Fifteen studies reported on ROSC after or during REBOA inflation. Of these fifteen articles, seven were (partially) from the same facility and therefore potentially enfold an overlap of patients. The occurrence of ROSC ranged from 18.2% to 75% (excluding small case series with less than ten patients). The largest study that commented on ROSC, as shown by Table 1 is by Romagnoli et al. and shows 62.7% had ROSC after REBOA inflation [27]. Three different papers, also from Baltimore, Maryland, reported ROSC in similar amount of patients [22, 28, 29]. Another study from Baltimore, Maryland reported ROSC in 18.2% of patients [30]. Others also reported successful ROSC ranging from 42.9% to 100% after REBOA placement in small case series of patients in TCA [21, 23, 25, 26, 31–35]. As can be seen in Table 1, all 15 articles specifically noted REBOA was used during cardiac arrest, not before.

### Survival

#### Survival without time definition

Eight of 26 articles reported survival rates of patients in TCA that received REBOA, without giving any time definition. Survival in these studies ranged from 0% to 36.6%. In two small case series, survival was 0% [32, 36]. In two retrospective cohorts that used data from the same database, survival rates were 36.6% and 27% in patients in TCA, but both studies did not mention explicitly that REBOA was inflated during arrest [24, 37]. Another study from this registry that included patients during the same time period did specify that REBOA was used during arrest and revealed a survival rate of 14%, without defining the length of survival [38].

#### Short-term survival

Four studies from the Shock and Trauma Center in Baltimore, Maryland commented on short-term survival. These studies reported similar numbers, ranging from 36% to 45.5% survival past ER/OR, 8-h survival and 12-h survival [22, 30, 39, 40]. 24 h survival was investigated by two studies, with overlap, which was 27% and 20% [30, 41]. Research without any overlap in patients showed a 40% 24-h survival in patients in TCA [42].

#### Long-term survival

In-hospital survival was examined by four studies that revealed a wide range of numbers, 42.9%, 19%, 8.7% and 0% [28, 31, 43, 44]. Survival to discharge ranged from 3.5% to 12.1%. The article that examined survival after TCA and REBOA with the most extensive number of patients was a study by Norii et al. The study from Japan included 175 patients in TCA receiving REBOA, of which 5.1% survived to discharge [45]. Survival to discharge was also reported by three other studies that could potentially have overlap in patients that revealed numbers ranging from 5.8% to 12.1% [27, 29, 39, 40]. These studies were performed in the same center during the same time period. Two other independent articles that show no overlap of patients with other studies, report a survival-to discharge of 3.5% and 10% in TCA patients [34, 46].

## Discussion

This scoping review summarizes the existing literature reporting the potential effect of REBOA on hemodynamics, ROSC and survival in patients in traumatic cardiac arrest. The congregated observational data in this study shows that REBOA seems to increase SBP in patients that receive REBOA in TCA and that there might be faster achievement of ROSC and potential survival benefit, but this needs to be researched more extensively and cannot be concluded from this review due to heterogeneity in design and data of the included studies. This scoping review included case reports, case series and retrospective cohort studies with a small number of patients in TCA, which results in an inability to draw any firm conclusion about the beneficial effect of REBOA in TCA.

A recently published review describes a clinical algorithm that clarifies whether to use REBOA or emergency thoracotomy in TCA patients. REBOA is foremostly indicated in patients in TCA with blunt or penetrating non-thoracic injury. While gaining access to the CFA, source of hemorrhage is located through thoracic X-ray, bilateral thoracostomy, eFAST and pelvic X-ray examination according to ATLS guidelines. In suspected thoracic injury, emergency thoracotomy is indicated as it offers access to intrathoracic bleeding and supradiaphragmatic crossclamping. For the latter, i.e., aortic occlusion, zone I REBOA could be regarded as an alternative to crossclamping. In traumatic cardiac arrest with ongoing CPR, REBOA was suggested to be inflated to zone I during CPR regardless if the suspected source of bleeding is abdominal, pelvic or retroperitoneal or when the source is unknown [47].

Measuring an accurate SBP in patients in TCA is a challenge since pressures under 30–40 mmHg are often undetectable by non-invasive blood pressure measurement (NIBP) and arterial line placement will not be a priority especially in case of a non-palpable radial or femoral artery. However, upon placement some REBOA catheters do have an arterial line port that measures blood pressure during balloon inflation, which could allow central aortic pressure when placing the REBOA. In the specific situation of ongoing CPR in traumatic cardiac arrest, chest wall compressions will likely impair a reliable blood pressure measurement, despite a central arterial line location.

As has been hypothesized, REBOA could be functional during (impending) cardiac arrest by improving coronary perfusion pressures which increases the chance at ROSC, which was confirmed in several animal studies [48–50].

If return of circulation could be achieved after REBOA deployment, it offers foremost an opportunity for additional interventions, but might also open the door to organ donation [22, 31]. In recent studies with large cohorts of patients in TCA, where other interventions than REBOA were implemented, ROSC ranged from 16% to 49% in, respectively, pre-hospital patients and patients that were resuscitated in the ED [51–54]. In the current review on REBOA for patients in TCA the occurrence of ROSC ranged from 18.2% to 75%, in which the majority of the studies was above 60%. The one with the lowest amount of ROSC, 18.2%, included only 11 TCA patients with very strict inclusion criteria, such as having an arterial line placed during arrest and could therefore have lower outcomes [30]. All articles that commented on ROSC, noted specifically that REBOA was inflated during cardiac arrest. This suggests that REBOA could have helped to achieve faster ROSC. In unique articles (without any potential overlap of patients), ROSC was 43%, 59%, 60%, 66.6% and 75% (excluding case reports) [24, 29, 31, 33, 34]. This might indicate a potential benefit of REBOA, compared to the below 50% rate of ROSC in the pre-REBOA era. More robust prospective and comparative series are needed to further define the attributive benefit of REBOA in the resuscitation of patients in traumatic cardiac arrest.

Survival rates of patients in extremis are generally low. REBOA is hypothesized to be of more influence on short-term survival, as it is supposed to function as a bridge to damage control surgery and/or angioembolization. Eight of 26 articles did not mention any time definition with their survival outcome numbers (0–36%). As a result, it is difficult to conclude anything from these percentages. In this review, 8-h survival was 40% and 12-h survival 36% [22, 30]. An included study by Teeter et al. investigated the difference in effect of REBOA and ET on patients in TCA. They discovered a higher survival past the emergency department (ED), 40.9% for REBOA patients and 10.7% for ET patients. This could partly be attributed to selection bias, as patients receiving ET were generally in worse condition, but might suggest a potential benefit REBOA could have on short-term survival in TCA patients [39]. Teeter et al. also examined the total duration of interruptions during CPR and compared REBOA to ET and revealed that total duration of interruptions during CPR was significantly less for patients undergoing REBOA. This could indicate REBOA would be more beneficial to use during cardiac arrest, as it is of much less surgical impact and CPR can be maintained more continuously.

Survival to discharge and 30-day survival are generally very low for patients suffering from out-of-hospital TCA and for patients arriving with ongoing CPR. Previous studies revealed a very low survival to discharge rate (0%–4.5%) for patients in TCA, where ET was most commonly used as an ultimate attempt [55–58]. Studies from 2006 and 2007 described a 7.5% survival to discharge rate in large cohorts of severely injured patients in TCA, and another more recent retrospective evaluation revealed a 30-day survival rate of 7.5% [59–61]. In this review, survival to discharge for TCA patients that underwent REBOA in addition to typical resuscitation methods ranged from 3.5% to 12%. Therefore, whether REBOA benefits survival to discharge in patients in TCA needs further evaluation and cannot be concluded based on the included studies due to heterogeneity, selection bias and lack of comparative studies.

This review is limited by a presumable overlap of patients, since several studies were performed in the same centers with overlapping inclusion periods. Due to the paucity of studies on this subject, these articles were however incorporated in the review, since all studies often commented on different hemodynamics that were useful to include in our analysis. Another limitation this review shows is the uncertainty of the exact time REBOA was deployed in the included articles. To thoroughly answer the question if REBOA alone provides a benefit to achieve ROSC, the time of REBOA inflation should be at the time the patient is in arrest, not before or after. In several articles, there was no specific statement that arresting patients had already achieved ROSC before balloon inflation or not. Additionally, it is assumable that, depending on the severity of injuries of the patients, patients received additional interventions besides REBOA as part of hemostasis and resuscitation.

The location of arrest of the patients in this review was divergent. Some articles only described patients arresting in the pre-hospital setting, some only described ED-resuscitations, and some both. This could be of influence on ROSC and survival outcome. Moreover, patients that were dead on scene were excluded in majority of studies, including possible survival bias.

Extensive studies have been performed regarding the effect of REBOA for patients in hemorrhagic shock, but not on patients in extremis, i.e., traumatic cardiac arrest. Further research is necessary for clarifying whether REBOA actually contributes to the resuscitation of trauma patients in cardiac arrest. Potential benefits are improved quality of cardiac compressions with reduced interruptions, avoiding thoracotomy with concomitant surgical trauma affecting the patients’ physiology, increasing coronary perfusion by increasing cardiac pre- and afterload and relatively limited resources needed by a trained physician which can be either a surgeon, emergency doctor or interventional radiologist.

Important to highlight is that especially short-term survival as an outcome could be more helpful to measure than survival to discharge, since REBOA is supposed to function as a bridge to further interventions, not as a definitive resuscitation method. Future studies should include data about the exact time of arrest and balloon inflation, to help define if ROSC occurs more rapid after using REBOA. Moreover, in order to discriminate the attributive effect of REBOA during TCA, detailed data regarding additional performed procedures that may influence rates of ROSC and survival, as well as trauma mechanism, zone of REBOA deployment, location of arrest and interference with CPR are necessary to describe in further research to draw any firm conclusion.

## Conclusion

This scoping review of studies with low level of evidence suggests that REBOA for patients in traumatic cardiac arrest might increase hemodynamic parameters, the occurrence of ROSC and decrease mortality. Due to a severe heterogeneity of studies, no firm conclusion can be drawn from the data presented in this scoping review. Future (prospective) studies should focus on retrieving more information about location of arrest, time of balloon inflation, ROSC and short- and long-term survival numbers.

## Supplementary Information

Below is the link to the electronic supplementary material.

Supplementary file1 (DOCX 15 KB)
