# Supplementary material for: The role of REBOA in patients in traumatic cardiac arrest subsequent to hemorrhagic shock: a scoping review
Source: Eur J Trauma Emerg Surg. 2022 Nov 6;49(2):693–707. doi: 10.1007/s00068-022-02154-z (PMC10175493; doi:10.1007/s00068-022-02154-z)
Supplement: Supplementary file 1 — Supplementary file1 (DOCX 15 KB) [file 68_2022_2154_MOESM1_ESM.docx]

**Supplementary Information**

**Supplementary information S1**

### History PubMed September 15, 2021

| Search | PubMed Query – September 15, 2021 | Items found |
| --- | --- | --- |
| #1 | "reboa"[tiab] OR "resuscitative endovascular balloon occlusion"[tiab] OR (("Balloon Occlusion"[MeSH] OR "balloon occlusion*"[tiab] OR "balloon tamponade*"[tiab] OR "balloon embolization*"[tiab] OR "balloon embolisation*"[tiab]) AND ("Resuscitation"[MeSH] OR "resuscitative"[tiab] OR "resuscitation*"[tiab]) AND ("Endovascular Procedures"[MeSH] OR "endovascular"[tiab]) AND ("Aorta"[MeSH] OR "aorta*"[tiab])) | 566 |

### History Embase.com September 15, 2021

| Search | Embase.com Query – September 15, 2021 | Items found |
| --- | --- | --- |
| #1 | 'resuscitative endovascular balloon occlusion'/exp OR reboa:ab,ti,kw OR ‘resuscitative endovascular balloon occlusion’:ab,ti,kw OR (('balloon occlusion'/exp OR (balloon NEAR/3 (occlusion* OR tamponade* OR embolization* OR embolisation*)):ab,ti,kw) AND ('resuscitation'/exp OR resuscitative:ab,ti,kw OR resuscitation*:ab,ti,kw) AND ('endovascular surgery'/exp OR endovascular:ab,ti,kw) AND ('aorta'/exp OR aorta*:ab,ti,kw)) | 681 |

### History Web of Science Core Collection September 15, 2021

| Search | Web of Science Core Collection Query – September 15, 2021 | Items found |
| --- | --- | --- |
| #1 | TS= (reboa OR “resuscitative endovascular balloon occlusion” OR ((balloon NEAR/3 (occlusion* OR tamponade* OR embolization* OR embolisation*)) AND (resuscitative OR resuscitation*) AND endovascular AND aorta*)) | 648 |
